# Correlation analysis of the mRNA and miRNA expression profiles in the nascent synthetic allotetraploid *Raphanobrassica*

**DOI:** 10.1038/srep37416

**Published:** 2016-11-22

**Authors:** Bingyuan Ye, Ruihua Wang, Jianbo Wang

**Affiliations:** 1 State Key Laboratory of Hybrid Rice, College of Life Sciences, Wuhan University, Wuhan 430072, China

## Abstract

*Raphanobrassica* is an allopolyploid species derived from inter-generic hybridization that combines the R genome from *R. sativus* and the C genome from *B. oleracea* var. *alboglabra*. In the present study, we used a high-throughput sequencing method to identify the mRNA and miRNA profiles in *Raphanobrassica* and its parents. A total of 33,561 mRNAs and 283 miRNAs were detected, 9,209 mRNAs and 134 miRNAs were differentially expressed respectively, 7,633 mRNAs and 39 miRNAs showed ELD expression, 5,219 mRNAs and 57 miRNAs were non-additively expressed in *Raphanobrassica*. Remarkably, differentially expressed genes (DEGs) were up-regulated and maternal bias was detected in *Raphanobrassica*. In addition, a miRNA-mRNA interaction network was constructed based on reverse regulated miRNA-mRNAs, which included 75 miRNAs and 178 mRNAs, 31 miRNAs were non-additively expressed target by 13 miRNAs. The related target genes were significantly enriched in the GO term ‘metabolic processes’. Non-additive related target genes regulation is involved in a range of biological pathways, like providing a driving force for variation and adaption in this allopolyploid. The integrative analysis of mRNA and miRNA profiling provides more information to elucidate gene expression mechanism and may supply a comprehensive and corresponding method to study genetic and transcription variation of allopolyploid.

Polyploidization, an ancient and ongoing evolutionary process, is a pervasive phenomenon in all angiosperm and played an important role in plant evolution through genomic merging and doubling^1^,^2^,^3^,^4^. Polyploids are typically classified as autopolyploids (duplication of the genome of a single species) and allopolyploids (duplicated combination of divergent genomes through hybridization). In fact, allopolyploid plays an important role in plant diversification and remains a significant speciation process today^3^,^5^, and many important agricultural plants; including wheat (*Triticum aestivum*), cotton (*Gossypium hirsutum*) and rapeseed (*Brassica napus*), have shown their hallmarks of allopolyploidy. Allopolyploid plants often show enhanced adaptability and arresting traits compared with their progenitors, which may contribute to crop domestication and natural selection^6^. Studies had reported a common occurrence in newly formed allopolyploids, which might cause genetic, epigenetic and transcriptional changes^3^,^5^, such as gene conversion events^7^,^8^ and deletions^4^,^9^,^10^, epigenetic changes, including DNA methylation^11^,^12^, and small RNA regulation^13^,^14^. Altered expression patterns^14^,^15^ and ELD (expression level dominate)^4^ have been reported as prominent phenomena in allopolyploids. Furthermore, recent studies on allopolyploidy have focused on ‘transcriptomic shock’: a rapid change in gene expression, reflecting non-additive gene expression from the predicted null hypothesis^16^, which has become a widespread phenomenon in newly formed allopolyploids. The ‘transcriptomic shock’ resulting from non-additive expression has recently been recognized as a crucial immediate outcome of allopolyploidy^17^,^18^,^19^,^20^,^21^,^22^.

Allopolyploidization is intriguing because it involves two processes: hybridization and whole genome doubling (WGD). Previous studies on allopolyploids have revealed that hybridization alters ancestral expression patterns immediately, massively, and irregulary^23^. Notably, hybridization between species is the driving force of genome evolution and new species formation. In fact, hybridization and WGD are often jointly involved in natural settings, resulting in growth vigour and phenotypic variation^24^. To study the effects of these two indispensable processes in allopolyploids, various results have been obtained from different plant taxa. Hybridization markedly affects Arabidopisis, but few variations have been associated with WGD^25^. Some reports have demonstrated that transcriptomic shock generates a modified pattern of WGD in *Senecio*; however, this effect cannot be explained by transcriptional changes^26^. Moreover, a semblable effect of both hybridization and WGD was observed in cotton^27^, while an oppositional effect of hybridization and WGD also has been reported in *Tragopogon*^18^. In addition, Xu showed that WGD has a distinct effect on gene expression and DNA methylation changes in synthesized *B. napus*^28^. The forgoing studies involved in hybridization and WGD coaction in interspecies hybrid allopolyploids have supplied valuable information. However, contrasting models have explained the respective effect of hybridization and WGD upon genomic and trancriptomic changes in inter-generic hybrid allopolyploids. Further investigations in inter-generic hybrid allopolyploids could increase the current understanding of the transcriptomic alterations that depend on hybridization and WGD.

Small RNAs (sRNAs) are endogenous single-stranded non-coding RNAs of approximately 21–25 nt in length including microRNAs (miRNAs) and small interfering RNAs (siRNAs), which regulate gene expression at the post-transcriptional level through complementary base pairing^29^,^30^ and epigenetic modifications^31^. MiRNAs often negatively regulate gene expression by promoting the degradation or suppressing the translation of target mRNAs. For example, miRNA regulation has been oberved in pathogens^32^, in response to stress, such as drought^33^,^34^ and extreme temperatures^35^,^36^. Wang suggested that miRNAs preferentially repress *A. thaliana* homologous loci when using the former as the maternal parent^37^. These observations demonstrate that miRNAs may serve as buffers against genomic shock and regulate DNA methylation. Despite these efforts, little attention has been paid to understanding how miRNA and mRNA expression is integrated into a dynamic and complex regulatory network, which might alter gene expression regulation and contribute to the enhancement of adaptation in inter-generic hybrid allopolyploids.

Studies on artificially synthesized polyploids and their progenitors are needed to understand the effects of polyploidization. The genus *Brassica* is a useful and importent model for agriculture and human nutrition^38^. *Raphanus sativus* (2n = 18, RR) is a valuable vegetable species that has contributed to human health and nutrition for hundreds of years and comprises multiple distinct cultivar terms with diverse morphological and phytochemical attributes. *Brassica oleracea* var. *alboglabra* (2n = 18, CC) is native to southern China, as a nutritious vegetable in *Brassica* plants. The name of this amphidiploid, *Raphanobrassica* (2n = 36, RRCC), reflects a combination of *Raphanus* and *Brassica*^39^. The tetraploid *Raphanobrassica* plant might be an ideal model to study gene expression dynamics and the corresponding genetic and epigenetic regulation in allopolyploids of inter-generic origins.

In this context, it is important to examine the regulation of gene expression in polyploids. Insights into miRNA-mRNA networks are necessary to understand the modulation of gene expression at the post-transcriptional level. However, the modulations that occur in the artificially synthesized tetraploid *Raphanobrassica* at the gene expression level and the interactions between miRNAs and mRNAs for the regulation of gene expression remain unknown. High-throughput sequencing technology offers unparalleled opportunities to address such issues and contributes to the current understanding of the molecular mechanisms that may contribute to adaption in the tetraploid *Raphanobrassica*. Thus, we executed mRNA and miRNA analyses of the leaves of *R. sativus, B. oleracea* var. *olablagra* and *Raphanobrassica* using RNA-Seq. The abundance data generated from gene expression and miRNAs analysis might suggest a RNA-mediated regulatory mechanism for homologous expression that may be essential for the tetraploid *Raphanobrassica* and its parents.

## Results

### Analysis of mRNA sequencing data

**Figure 1:**
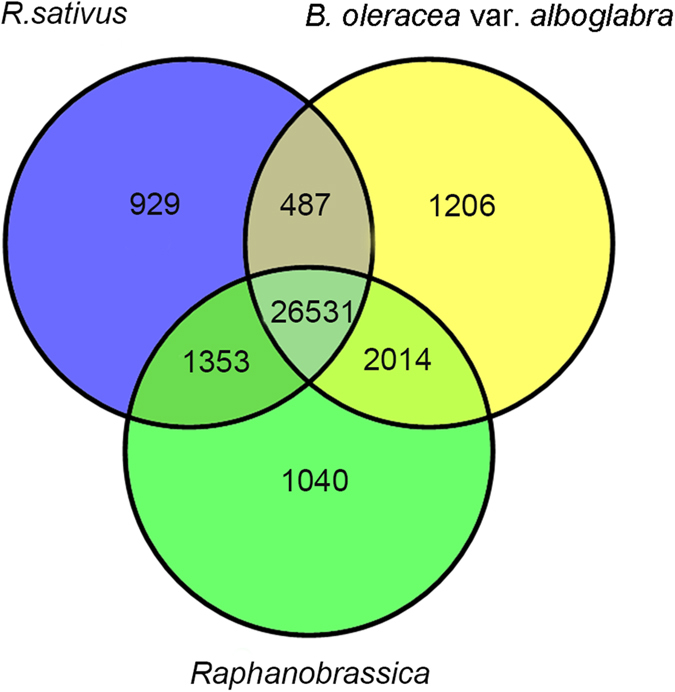
Venn diagram shows genes expressed in *Raphanobrassica* and its parents. A, *Raphanus sativus*; B, *Brassica oleracea* var. *alboglabra*; C, *Raphanobrassica*.

After employing the RNA-seq (modified) method, an average of 20 million reads were detected in each library, and 84.03%, 86.85%, and 86.22% clean reads could be individually mapped to reference genomes in the *R. sativus, B. oleracea* var. *alboglabra*, and *Raphanobrassica* libraries, respectively (Table 1). In the present study, a total of 33,561 genes were detected in *Raphanobrassica* and its parents, and analysis of the gene coverage distribution showed that 14,875, 18,454 and 18,628 genes had more than 90% coverage in *R. sativus, B. oleracea* var. *alboglabra* and *Raphanobrassica*, respectively (Fig. S1), indicating that a significant number of genes are satisfactorily matched, and the recent Abecedarian data are usable for further studies. Venn diagram shows visible gene expression in *R. sativus, B. oleracea* var. *alboglabra*, and the tetraploid *Raphanobrassica* (Fig. 1). A total of 26,531 genes were co-expressed in the tetraploid *Raphanobrassica* and its parents. Furthermore, 929 genes (*R. sativus*), 1,206 genes (*B. oleracea* var. *alboglabra*), and 1,240 genes (tetraploid *Raphanobrassica*) genes were specifically expressed.

### Gene ontology (GO) enrichment annotation analysis of all detected genes

In the present study, 28,314 genes (84.36%) among the 33,560 detected genes had at least one GO annotation. All matched relevant genes were classified into 3 functional categories: molecular functions, biological processes and cellular components, including 44 terms (Fig. S2). These genes were classified into 23 terms in the biological process category, while 12 terms were matched in the molecular function category, and 9 terms were matched in cellular component category. The terms ‘cell’ and ‘organelle’, which included 19,096, and 12,526 genes, were dominant in cellular component category. In the molecular function category, the terms ‘binding’ and ‘catalytic activities’ were most significantly enriched, with 13,249 and 11,547 genes, respectively. There was also enrichment for the terms in response to ‘biological process’, ‘categories’, ‘cellular process’ and ‘metabolic process’, indicating that *Raphanobrassica* and its parents underwent exquisite metabolic activities, suggesting further exploration of additional mechanisms that relevant to metabolic activities in polyploids.

### Differentially expressed genes (DEGs) between *Raphanobrassica* and its parents with GO functional categories analysis

**Figure 2:**
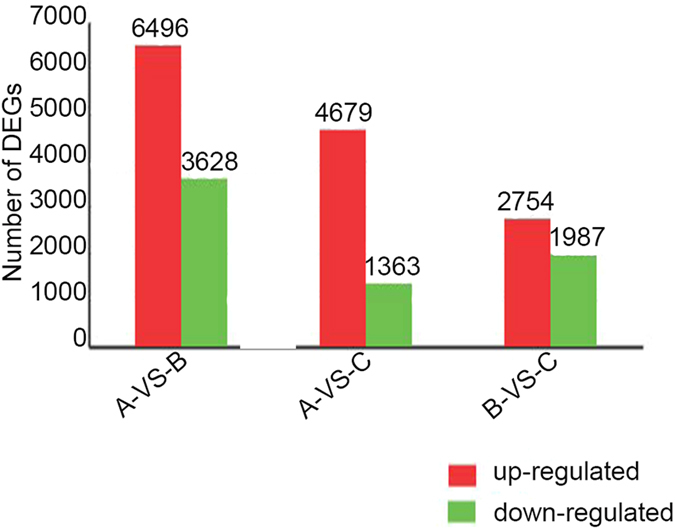
Differentially expressed genes detected between *Raphanobrassica* and its parents. The number of up-regulated and down-regulated genes between B, *B. oleracea* var. *alboglabra* and A, *R. sativus*, C, *Raphanobrassica* and A, *R. sativus*, C, *Raphanobrassica* and B, *B. oleracea* var. *alboglabra* are revealed.

A total of 9,209 genes were differentially expressed (log_2_Ratio ≥ 1, p ≤ 0.05) between tetraploid *Raphanobrassica* and its parents (Fig. 2), and 6,042 genes were significantly differentially expressed between tetraploid *Raphanobrassica* and *R. sativus*, among these, 4,679 genes were up-regulated and 1,363 genes were down-regulated in the tetraploid *Raphanobrassica*. Moreover, 4,741 genes were differentially expressed between *Raphanobrassica* and *B. oleracea* var. *alboglabra*, with 2,754 up-regulated and 1,987 down-regulated genes in *Raphanobrassica*. These data suggested that differentially expressed genes are up-regulated in *Raphanobrassica* compared with its parents and there were more differentially expressed genes between *Raphanobrassica* and its maternal parent *R. sativus.*

**Figure 3:**
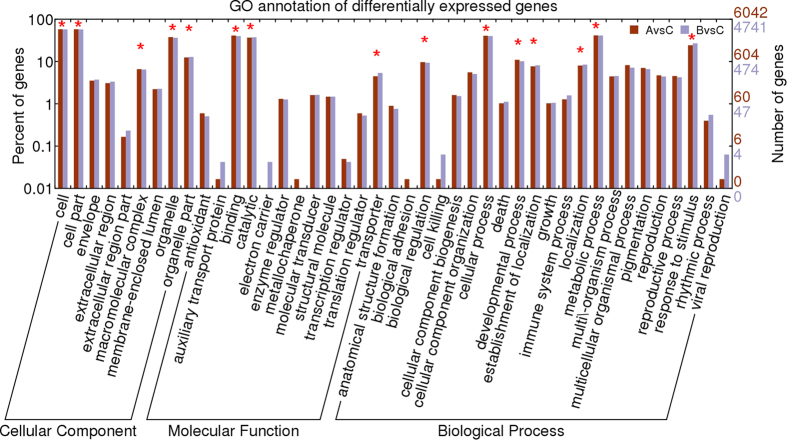
Functional annotation of different express genes between *Raphanobrassica* and its parents based on GO terms.

To examine the gene expression differences between *Raphanobrassica* and its parents, we performed a GO enrichment analysis and categorized the DEGs to the secondary GO terms classification. A total of 9 terms belonged to the cellular component category, 12 functional terms belonged to the molecular function category and 22 functional terms belonged to the biological process category (Fig. 3). In the cellular component category, the terms ‘cell’, ‘cell part’, ‘organelle’ ‘organelle part’ and ‘macromolecular complex’ were significantly enriched, and the term ‘organelle part’ was more significantly expressed in contrast with the other three terms in the *Raphanobrassica/B. oleracea* var. *alboglabra* comparison for the cellular component category. The terms ‘binding’, ‘catalytic’ and ‘transporter’ were dominant in the molecular function category, with ‘binding’ biased to the comparison *Raphanobrassica/R. sativus;* however, the terms ‘catalytic’ and ‘transporter’ were biased to paternal parent *B. oleracea* var. *alboglabra.* ‘Cellular process’, ‘metabolic process’ and ‘response to stimulus’ were the highest three GO terms significantly enriched in the biological process category. Interestingly, the terms ‘developmental process’, ‘establishment of localization’, ‘biological regulation’ and ‘localization’ were slightly lower but also significantly enriched in the biological process category. In this category, ‘biological regulation’, ‘cellular process’, ‘developmental process’ and ‘metabolic process’ were biased to the maternal parent *R. sativus*, and the terms ‘establishment of localization’ and ‘response to stimulus’ were biased to the paternal parent *B. oleracea* var. *alboglabra.* These results imply that tetraploid *Raphanobrassica* was distinct reflecting a complex process of expression.

### KEGG analysis of DEGs between *Raphanobrassica* and its parent

After mapping to the reference canonical pathways, the 9,209 DEGs (Q value ≤ 0.05) detected from *Raphanobrassica* and its parents were assigned to 127 pathways. In the comparison *R. sativus/Raphanobrassica*, ‘biosynthesis of secondary metabolites’, ‘metabolic pathway’, ‘plant-pathogen interaction’ and ‘plant hormone signal transduction’ were significantly enriched. In the comparison of *B. oleracea* var. *alboglabra/Raphanobrassica*, the genes were significantly enriched in the ‘metabolic’, ‘plant-pathogen interaction’ and ‘plant signal transduction’ pathways. The top 10 (Table S1) pathways showed that the high gene enrichment was sorted in order of gene numbers in *Raphanobrassica* compared with its parents, with the terms ‘metabolic pathways’, ‘biosynthesis of secondary metabolites’, ‘plant-pathogen interaction’ and ‘plant hormone signal transduction’. The metabolic pathway contained the largest gene numbers (1,716) among all detected pathways, while the pathway ‘ribosome’, containing 180 genes, showed the highest genes expression levels among all detected KEGG pathways. Thus, some remarkable gene expression changes might occur for one or more of these genes.

### Analysis of differentially expressed genes among putative transcription factor genes

Using the rape transcription factor database regarding the identified DEGs, 3,387 genes were associated with 63 transcription factor families, and the expressions of the genes in most of these families was down regulated. The highest gene expression family was ‘bHLH’, with an average RPKM value of 1,870 (total amount divided by the number of genes expressed), and the second highest gene expression family was ‘MYB’, with an average expression quantity of 1,705. ‘AP2-EREBP’ and ‘C3H’ were the third and fourth highest gene expression families, possessing 1,525 and 1,225 average expression quantities, respectively. The last gene expression family, ‘bZIP’ had a gene expression quantity of 1,150 (Fig. S3). The highest expressed family in the present study, ‘bHLH’, has a reported function of transcriptional activation of gene expression under drought stress in Arabidopsis, and growth-promoting bHLH degradation by inhibition shade avoidance based on a phytochrome-mediated process. This result suggests that the family ‘bHLH’ might also be a nonnegligible force, played an important role for variation and adaptation in polyploidy.

### Identification of non-additively expressed genes in the tetraploid *Raphanobrassica* relative to its parents

**Figure 4:**
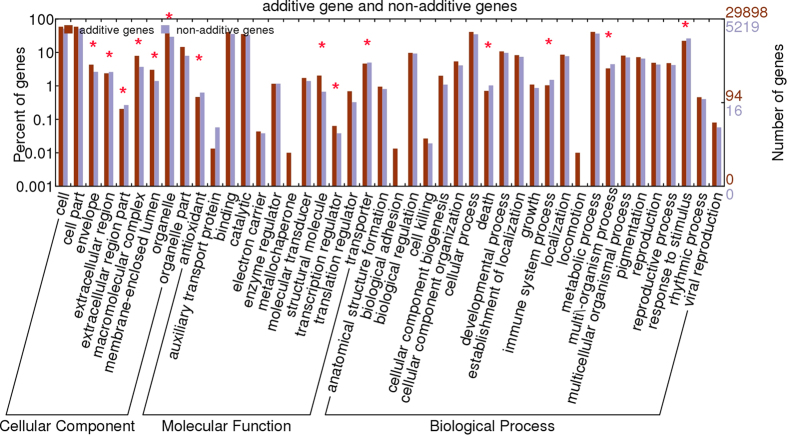
Functional annotation of non-additive genes and additive genes in tetraploid *Raphanobrassica* based on GO terms. Non-additive genes and additive genes are grouped to the secondary classification of GO terms.

To determine non-additively expressed genes, we compared gene expression levels in *Raphanobrassica* with the average genes expression values in the parents (MPVs). If the value of a gene in *Raphanobrassica* showed at least a two-fold change and P < 0.05 relative to the MPV, then the gene was regarded as non-additive expression^37^. A total of 5,219 genes, accounting for 15.55% of the total detected genes, were non-additively expressed in the tetraploid *Raphanobrassica*. Among these non-additive genes, 2,280 genes were up-regulated and 2,932 genes were down-regulated in the tetraploid *Raphanobrassica* compared to its parents. We observed additive gene expression for a majority of the expressed genes in *Raphanobrassica*, whereas non-additively expressed genes represented only a small portion of the expressed genes. To further explore the difference in the functional category distribution between additively and non-additively expressed genes, we assigned secondary classification GO terms. The genes belonged to 9, 12 and 24 functional terms in cellular component, molecular function and biological process, respectively. In the cellular component category, the number of additive and non-additive gene significantly differed for the functional terms ‘membrane-enclosed lumen’, ‘organelle part’, ‘extracellular region’, ‘macromolecular complex’ and ‘envelope’ and showed significant enrichment in additive genes compared with non-additive genes except ‘extracellular region’. In the molecular function category, the term ‘structural molecule activity’ and ‘translation regulator’ showed significant differences in gene numbers between additive and non-additive gene, and the terms ‘antioxidant’ and ‘transporter’ were significantly enriched with non-additive genes predominant. In the biological process category, four functional terms, ‘death’, ‘immune system process’, ‘multi/-organism processes’ and ‘response to stimulus’ had significantly more non-additive than additive genes. The non-additive genes were belonged to 9, 11 and 21 terms in cellular component, molecular function, and biological process, respectively (Fig. 4). More than 40% of the non-additively expressed genes were enriched in ‘cell’, ‘cell part’ and ‘organelle’ in the cellular component category, while ‘catalytic activity’, ‘binding’ and ‘transporter’ were significantly enriched in the molecular function category, and the terms ‘metabolic process’, ‘response to stimulus’ and ‘cellular process’ showed significant enrichment in the biological process category.

### Parental gene expression level dominance (ELD) in allotetraploid *Raphanobrassica*

**Figure 5:**
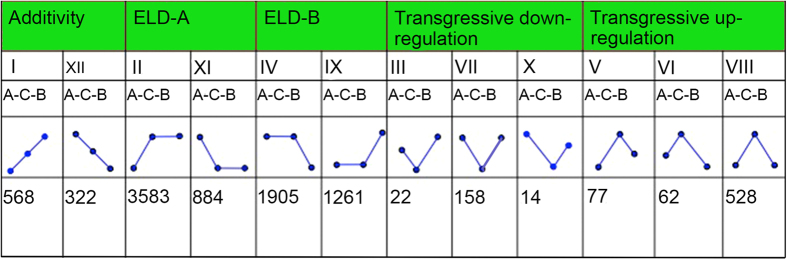
ELD of genes in tetraploid *Raphanobrassica*. A, *Raphanus sativus;* B*, Brassica oleracea* var. *alboglabra;* C, *Raphanobrassica*. ELD-a, genes with expression level similar to that in *R. sativus*; ELD-b, genes with expression level similar to that in *B. oleracea* var. *alboglabra*.

To accurately analyse the gene expression patterns between *Raphanobrassica* and its parents, we further classified all of the detected genes into 12 categories according to the methods of Rapp^22^ and Grover^40^. Among these, the differentially expressed genes in only 4 categories were detected between the parental lines and exhibited statistically similar levels of expression in *Raphanobrassica* to those in the parents, indicating parental expression level dominance (ELD) (Fig. 5). In the 4 categories, we designated genes with expression levels in *Raphanobrassica* that were statistically similar to those in *R. sativus* as ELD-a genes and those similar to *B. albaglabra* as ELD-b genes.

In *Raphanobrassica,* 7,633 genes showed parental ELD. The down-regulated genes (884) in ELD-a patterns showed relatively lower expression in *Raphanobrassica* compared with the up-regulated genes (3,583), and the genes in ELD-b did not show significant discrepancy ratios between up-regulated (1,905) and down-regulated (1,261) patterns. Generally, *Raphanobrassica* had relatively more ELD-a genes than ELD-b genes (4,467/3,166). Thus, the gene expression in *Raphanobrassica* displayed ELD bias towards the maternal progenitor *R. sativus.*

We further pursued the potential functions of the genes showing parental ELD in *Raphanobrassica*. The GO analysis of ELD-a genes (4,467) revealed the enrichment of genes for the biological processes ‘establishment of localization’, ‘metabolic process’, ‘localization’ and ‘cellular process’, and among the ELD-b genes in biological processes, three terms, ‘response to stimulus’, ‘metabolic process’, and ‘cellular process’, were enriched among the ELD-b genes in the biological process category (Fig. S4). In addition, the terms ‘establishment of localization’ and ‘localization’ were specifically enriched in ELD-a genes, and the ELD-b genes were significantly enriched for ‘response to stimulus’ after removing the two coexisting terms. In the molecular function category, the terms ‘catalytic activity’ and ‘binding’ coexisted in both ELD-a and ELD-b genes with the term ‘transporter activity’ and were specifically enriched in ELD-a genes.

### Identification of miRNAs and their target genes in *Raphanobrassica* and its parents

**Figure 6:**
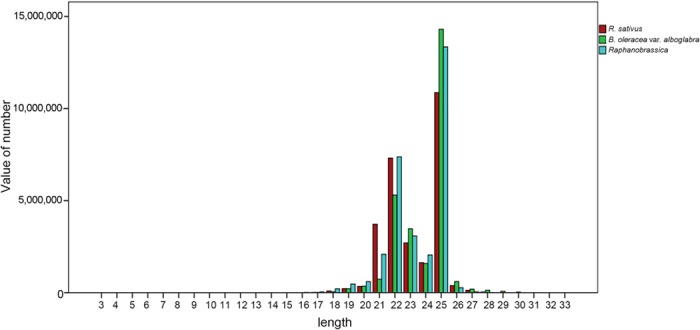
The length distribution and categories distribution of small RNAs in *R. sativus, B.* oleracea var. alboglabra, and *Raphanobrassica*.

A total of 85,593,286 small RNA sequencing reads were generated from three libraries. After removing adapter and structural RNA sequences, we identified 27,776,413, 27,290,301, and 30,093,748 clean reads within a range of 21–25 nucleotides from *R. sativus, B. oleracea var. alboglabra,* and *Raphanobrassica*, respectively. The reads distributed between 21–25 nt were consistent with the common features of sRNAs (Fig. 6), and 24 nt was the most abundantly enriched, accounting for 39.57% of clean reads in *R. sativus,* 45.07% in *B. oleracea* var. *alboglabra* and 52.75% in *Raphanobrassica*. In addition, the second most abundant read was 21 nt, accounting for 26.60% of clean reads in *R. sativus*, 19.54% in *B. alboglabra*, and 24.92% in *Raphanobrassica*.

Compared with the miRBase database, we generated 281 miRNAs in total, with 277 in *R. sativus*, 274 in *B. oleracea* var. *alboglabra* and 271 in *Raphanobrassica* (Fig. S5). Furthermore, 267 miRNAs were conserved in all three libraries, accounting for 95.01% of the 281 miRNAs. Subsequently, 3 miRNAs were co-expressed in *R. sativus* and *B. oleracea var. alboglabra*, and 7 miRNAs were co-expressed in *Raphanobrassica* and *R. sativus*, while 4 miRNAs were co-expressed in *Raphanobrassica* and *B. oleracea var. alboglabra*. Because a deep-sequencing approach was used to recover small RNA reads, the relative expression level of a specific miRNA can be measured from the frequency of its read count. Based on the abundance of miRNAs, we observed that the miRNAs expression levels were highly variable, from 1 to 784,100 sequence reads among the three libraries (Table 2). Specifically, 43.53% of miRNAs was observed for expression of less than 100 reads, while 20.64% of miRNA expression levels ranged from 100 to 500 reads, and 16.13% of those were between 1,000 and 5,000 reads. The percentage of miRNAs whose expression levels were more than 10,000 reads was 7.59%. For example, one of the most abundant (>10,000 reads) miRNAs in the three libraries was miR172, with 495,478 reads in *R. sativus*, 474,293 reads in *B. oleracea var. alboglabra,* and 632,699 reads in *Raphanobrassica*. Interestingly, one of the targets of miR172 is *AP2* gene, which is essential for regulation of leaf conversion from young to mature^41^. MiR166, miR167, miR172, and miR158 had more than 10,000 reads in all three libraries, with similar expression profiles. These data suggest that the abundant miRNAs in *Raphanobrassica* and its parents might involve in a number of key biological processes.

**Figure 7:**
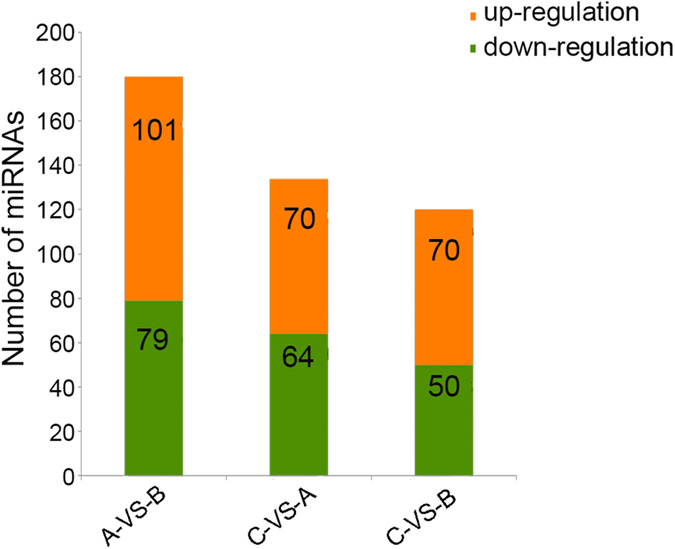
miRNAs detected between *Raphanobrassica* and its parents. The number of up-regulated and down-regulated miRNAs between *B. oleracea* var. *alboglabra* and *R. sativus, Raphanobrassica* and *R. sativus*, tetraploid *Raphanobrassica* and *B. oleracea* var. *alboglabra* are revealed.

Subsequently, compared with *R. sativus*, 134 significantly differentially expressed miRNAs (log_2_Ratio ≥ 1, p ≤ 0.05) were detected, with 64 up-regulated and 70 down-regulated miRNAs in *Raphanobrassica* (Fig. 7). In 120 differentially expressed miRNAs between *Raphanobrassica* and *B. oleracea* var. *alboglabra*, 50 up-regulated and 70 down-regulated miRNAs were detected in *Raphanobrassica*.

To identify the potential roles of target genes of differentially expressed miRNAs in *Raphanobrassica/R. sativus* and *Raphanobrassica/B. oleracea* var. *alboglabra,* a total of 182 differentially expressed miRNAs and 3,303 target genes were detected (Table S2), with 182, 179 and 182 miRNAs targeting 1,103, 1,098 and 1,102 genes in *R. sativus, B. oleracea* var. *alboglabra* and *Raphanobrassica*, respectively. In addition, 85 and 74 miRNAs with 394 and 478 target genes existed in *Raphanobrassica/R. sativus* and *Raphanobrassica/B. oleracea* var. *alboglabra*, respectively. Moreover, differentially expressed miRNA targets for *Raphanobrassica/R. sativus* and *Raphanobrassica/B. oleracea* var. *alboglabra* were placed in secondary GO terms classification to compare the difference of *Raphanobrassica* from its two parents (Fig. S6). In the high frequency of occurrence terms ‘organelle’ and ‘organelle part’, *Raphanobrassica* showed a significant difference from its paternal parent *B. oleracea* var. *alboglabra* than *R. sativus,* while in the conversed terms ‘cell’, ‘cell part’, ‘catalytic, ‘cellular process’ and ‘localization’, *Raphanobrassica* showed a consistent difference from its maternal parent *R. sativus.* The difference in the miRNA target expression between the progeny and its parents might reflect the different relationship types.

### ELD-miRNAs between *Raphphanobrassica* and its parents

**Figure 8:**
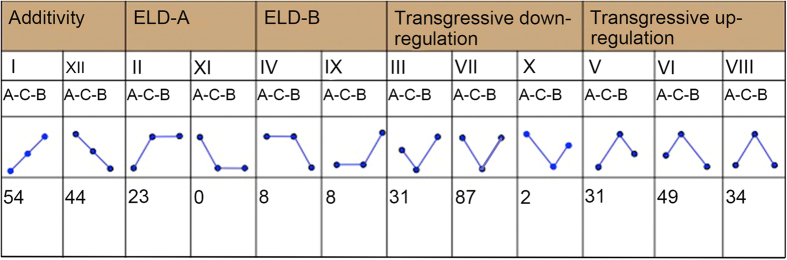
ELD of miRNAs in tetraploid *Raphanobrassica*. A, *Raphanus sativus;* B*, Brassica oleracea* var. *alboglabra;* C, *Raphanobrassica*. ELD-A, miRNAs with expression level similar to that in *R. sativus*; ELD-B, miRNAs with expression level similar to that in *B. oleracea* var. *alboglabra*.

We further divided all miRNAs into 12 patterns (Fig. 8) and observed that 39 miRNAs in *Raphanobrassica* were ELD-expressed compared with the MPVs, 23 miRNAs (pattern II and XI) exhibited ELD-a, and all ELD-a miRNAs were biased to up-regulated (pattern II). Interestingly, differentially expressed miRNAs in ELD-b were average, with 8 ELD-b miRNAs in pattern IV showing biased towards up-regulated patterns, and the other 8 ELD-b miRNAs showing down-regulated pattern IX. Furthermore, 120 differentially expressed miRNAs were transgressively down-regulated, while 114 differentially expressed miRNAs were transgressive up-regulated. Among the transgressive miRNAs, we identified 2 miRNAs with more deflection than the paternal parent among the transgressive down-regulated miRNAs in pattern X, but pattern III (biased to the maternal parent) conceived a comparatively high proportion (31 of 224) of transgressive down-regulated miRNAs. This result may illustrate the existence of maternal-bias miRNAs in *Raphanobrassica*. Overall, miRNAs displayed preferential pattern III in transgressive down-regulated miRNAs, likely reflecting the regulation of their target gene expression in *Raphanobrassica.*

### Comprehension of non-additive miRNAs and their target genes

Compared with MPV, 57 non-additively expressed miRNAs and 167 target genes were detected. To further explore the functional categories distribution of the non-additively expressed miRNAs target genes in *Raphanobrassica*, we assigned these genes to secondary classification GO terms (Fig. S7). In the cellular component category, cell and organelle were significantly enriched. The terms that include ‘binding’, ‘catalytic’, and ‘transporter’ were significantly enriched in molecular function category. ‘Biological regulation’, ‘cellular process’, ‘developmental process’, ‘localization’, ‘metabolic process’, ‘multicellular organismal process’, ‘pigmentation’ and ‘response to stimulus’ were significantly enriched in the coherent target genes of non-additive miRNAs.

### Correlation analysis of related target genes based on the mRNA-miRNA interaction network

**Figure 9:**
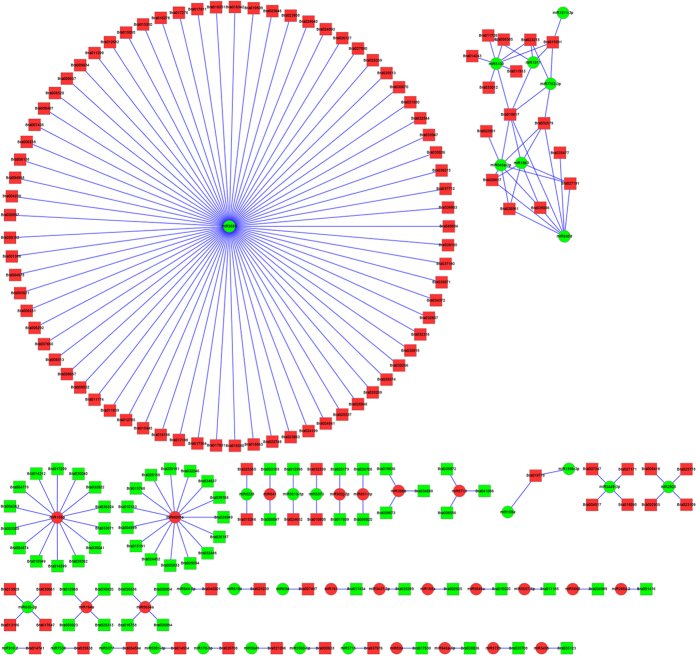
The interaction network of miRNAs combine with mRNAs. The circle and rectangle nodes represent miRNAs and mRNAs, respectively. The up-regulated and down-regulated nodes are separately colored in red and green.

**Figure 10:**
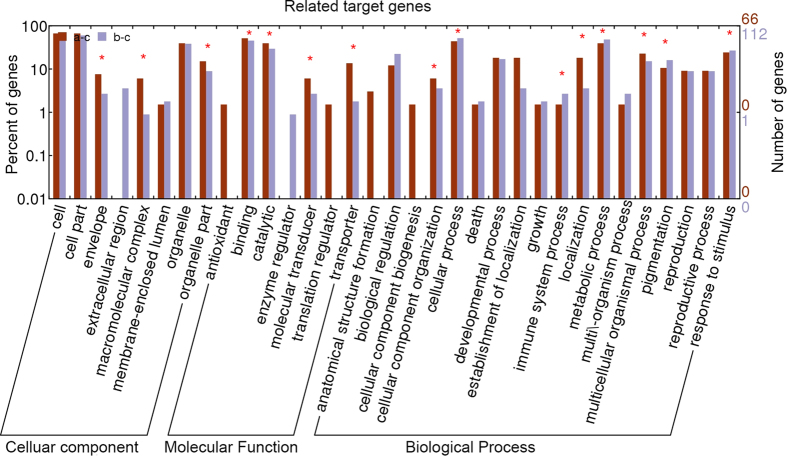
GO annotation of related target genes between *Raphanobrassica* and its parents. Negatively related genes from miRNAs were classifying to the secondary classification of GO terms separate compared between *Raphanobrassica* and *R. sativus, Raphanobrassica* and *B. oleracea* var. *alboglabra*.

We defined a predicted miRNA target gene when its expression pattern was in contrast with that of the miRNA, reflecting the fact that mRNA expression was negatively correlated with miRNA expression. Based on this rule, we identified a total of 178 related target genes in the two comparisons between the polyploidy and the two parents (Fig. 9), with 32 down-regulated and 62 up-regulated genes in *Raphanobrassica/R. sativus*, and 30 down-regulated and 82 up-regulated genes in *Raphanobrassica/B. oleracea var. alboglabra* retro-regulation by miRNAs. Among the related target genes, only 31 genes were non-additively expressed, targeting 14 miRNAs. Obviously, miRNA5658 contains a considerable number of related target genes, and 15.00% (12/80) related target genes targeted by miRNA5658 were non-additively expressed. Moreover, both miRNA156a and miRNA8005a have 14 related target genes, achieving the second and third most highly correlated miRNAs. Among the 14 genes targeted by miRNA156a, only bra014212 was non-additively expressed. In miRNA8005a, two genes, bra015748 and bra032246, were non-additively expressed. To screen these differentially expressed miRNAs target genes, we performed a functional enrichment analysis based on GO functional annotations. Interestingly, the term ‘metabolic process’ and ‘response to stimulus’ were significantly enriched in the relevant target genes of *Raphanobrassica/B. oleracea* var. *alboglabra* compared with *Raphanobrassica/R. sativus* (Fig. 10). As a result of frequently restrained miRNAs, the genes related to ‘metabolic process’ and ‘response to stimulus’ in *Raphanobrassica* were up-regulated compared with its parents. Based on the information contained in GO enrichment of mRNAs and miRNAs, the GO term ‘metabolic process’ was significantly enriched, no matter in differentially expressed mRNAs/miRNAs (target genes) or non-additively expressed mRNAs/miRNAs (target genes) in *Raphanobrassica* compared with its parents. MiRNA5658a, which contained 44.94% of miRNA-target pairs in correlation network in the present study, was also significantly enriched in the terms ‘protein catabolic process’, ‘response to stress’ and ‘response to biotic and abiotic stress’ in *Ocimum basilicum*^42^. The 31 related target genes showing non-additive expression were also significantly enriched in ‘response to stress’ and response to biotic’ in the present study, but the non-additively expressed gene targets of miRNA5658a were significantly enriched in the ‘metabolic process’ category (Fig. S8).

### qRT-PCR validation of significant differentially expressed mRNA-genes and miRNAs

**Figure 11:**
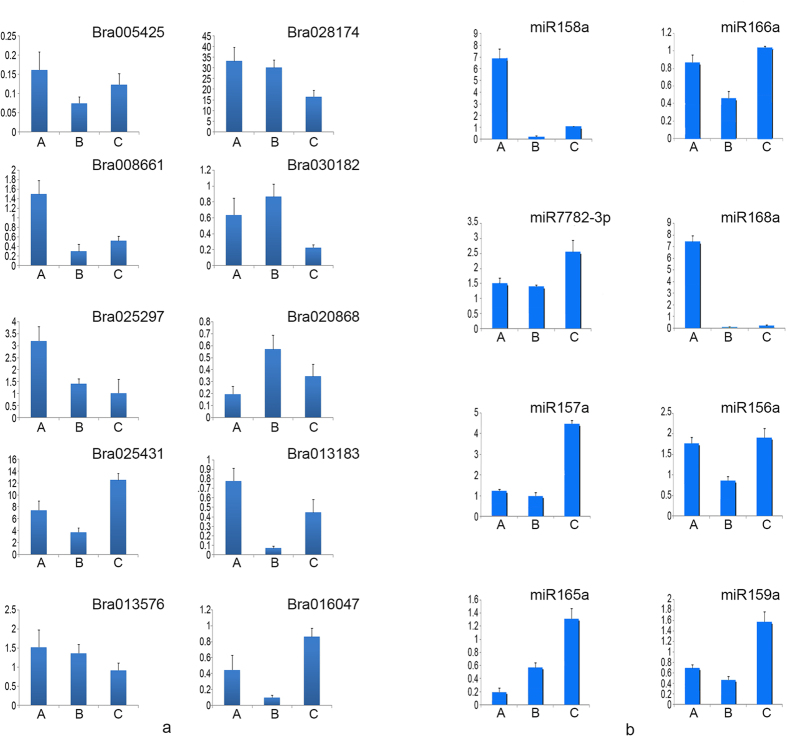
The expression level of selected differentially expressed mRNAs and miRNAs are validated by quantitative real-time RT-PCR.

To confirm the expression profiles obtained from high-throughput sequencing, stem-loop real-time quantification RT-RCR was performed to study the ten mRNAs and eight miRNAs (Fig. 11 and Table 3). The expression patterns of the selected miRNAs and miRNAs were consistent with those determined using high-throughput sequencing, indicating that the sequencing data produced in the present study were reliable and could be subjected to further analysis.

## Discussion

Until recently, many studies have examined the mechanism gene expression in polyploidy^4^,^14^,^15^,^20^. For example, Zhao^15^ suggested that high ploidy levels in the *Brassica* hexaploid might reflect increased genome and transcriptome changes. In Arabidopsis allopolyploids, small RNAs act as a buffer against genomic shock by analysing miRNA expression and siRNA distribution^35^. *Raphanobrassica* was artificially synthesized from modern cultivars of *R. sativus* and *B. oleracea* var. *alboglabra* without obvious reproductive barriers, indicating almost instant genetic stabilization. The advent of high-through sequencing technology provided unprecedented opportunities to examine this inter-generic hybridization allopolyploid with the data obtained from two pivotal components: mRNA and microRNA.

### Insight into gene expression changes in polyploids derived from inter-generic and interspecific hybridization

Non-additively expressed mRNAs and miRNAs in polyploidy have been detected in allotetraploid *Arabidopsis*, hexaploid wheat and citrus allopolyploids^4^,^29^,^42^. In the present study, the RNA-seq technique were used to compare mRNA and miRNA expression profiles between *Raphanobrassica* and its parents, and a total of 9,209 (27.43%) genes showed expression divergence, while 5,219 (15.55%) genes were non-additively expressed in the tetraploid *Raphanobrassica.* Interestingly, compared with the interspecific polyploids *Brassica* hexaploid in the study of Zhao^15^, 17.44% genes were differentially expressed, and only 7.79% genes were non-additively expressed in the allohexaploid. The frequency of changes in non-additive gene expression increased in the inter-generic tetraploid, compared with the *Brassica* hexaploid, likely reflecting higher gene expression divergence between the inter-generic parents. Among the miRNAs, 41.79% of miRNAs were non-additively expressed and differentially expressed between tetraploid *Raphanobrassica* and its parents, while 69.11% of miRNAs were non-additively expressed in the interspecific hybrid *Brassica* hexaploid^14^, which was obviously higher than that in the inter-generic tetraploid *Raphanobrassica*. Subsequently, a previous study reported that the volume of production was not increased following the ploidy level in *R. sativus* × *B. alboglabra* hybrids, with the tetraploid showed highest output among diploid, tetraploid, hexaploid and octaploid species^43^. The higher output in this tetraploid compared with diploid and other polyploid species may also reflect the increased frequency of changes in non-additive gene expression. Thus, inter-generic tetraploid *Raphanobrassica* may have great genomic and transcriptomic changes in mRNA expression.

The phenomenon of genes showing parent-biased has been frequently observed in many speices^4^,^6^,^37^. For example, Li^4^ showed that the parent-biased expressed genes were likely major contributors to key adaptation traits, such as stress tolerance and flexibility under flowering conditions. Del^44^ reported that some species show a maternal bias in mRNA transcripts and gene activity in early embryogenesis. However, a few studies also suggested that the contribution of maternal genomes had no visible distinction from the paternal genomes. Not surprisingly, a marked number of genes also showed parental-bias expression in inter-generic *Raphanobrassica.* We used ELD-genes to explain this phenomenon, and among the 9,209 differential expression genes between progeny and progenitors, a total of 4,467 (48.50%) genes belonged to ELD-a, while 3,166 (34.37%) genes belonged to ELD-b in *Raphanobrassica*, indicating that a relatively larger portion of genes showed biased expression to the maternal parent *R. sativus* in *Raphanobrassica*. In addition, model II (gene up-regulated in *Raphanobrassica* than in *R. sativus*, and relatively unaltered compared with *B. oleracea* var. *alboglabra*) was significantly higher than model XI (Fig. 5), suggesting that the genes in ELD-a exhibit increased rather than decreased expression level in the progeny, indicating that interesting variations in the gene expression might have occurred in this region. Moreover, all of the ELD-a miRNAs belonged to model II (miRNA up-regulated in *Raphanobrassica* than in *R. sativus*, and relatively unaltered compared with *B. oleracea* var. *alboglabra*), but miRNAs in the ELD-b showed equal distribution, indicating that if the progeny miRNAs showed no significant changes compared with the other parents, then the miRNAs that the maternal parent *R. sativus* transmitted to *Raphanobrassica* were down-regulated. However, the miRNA in *Raphanobrassica* transmitted from the paternal parent *B. oleracea var. alboglabra* were impartially changed in an up and down regulated mode. This result may suggest a comparatively specific difference between *Raphanobrassica* and its maternal parent *R. sativus.* These data, combined with the gene and miRNA expression analysis, showed that the directivity between *Raphanobrassica* and *R. sativus* was larger than the contrast between *Raphanobrassica* and *B. oleracea var. alboglabra.* The maternal biased was consistent with the previous conclusions of Zhao^15^ and Shen^14^ concerning the *Brassica* hexaploid.

### Correlation analysis of miRNAs and mRNAs are significant to study polyploidy

Recent studies have reported that the target genes repressed by miRNAs are typically involved in salt, cold or drought response treatments^4^,^44^, and 178 related target genes were detected in *Raphanobrassica,* and most of these target genes were significantly enriched in down-regulated compared with up-regulated related target genes, except the terms ‘macromolecular complex’, ‘molecular transducer’ and ‘response to stimulus’ (Fig. S9), suggesting that the related target genes exhibited an increased influence in most of the biological pathways in the progeny. In the interspecific hybrid allopolyploid *Brassica* hexaploid, only the term ‘response to stimulus’ was significantly enriched in the down-regulated miRNA target genes^14^. These results demonstrate that the related target genes generated from inter-generic hybridization might play an important role in the growth and development changes observed in the inter-generic *Raphanobrassica.*

Some studies have suggested that the miRNAs-mRNAs regulation pattern includes both the coherent and incoherent relationship^15^,^45^ and that one miRNAs may have diverse target genes. For example, a single miRNA ‘miR167a’ in this *Raphanobrassica* can target multiple mRNAs, including Bra004125, Bra010776, Bra028110, and Bra035727. Among these, some targets show up-regulated expression compared with miRNAs, while other targets show down-regulated expression compared with miRNAs. Indeed, miRNAs may have a certain specific target mRNA and contribute to the regulation of miRNA (for example, target Bra028668 can be regulated by miR162a in *Raphanobrassica*). However, target genes may be regulated by several up- and down-regulated miRNAs. Bra004674 can be passively regulated by up-regulated miR156a in *Raphanobrassica/B. oleracea* var. *alboglabra* and down-regulated miR156a in tetraploid *Raphanobrassica/R. sativus.* This consequence indicated the target genes that did not respond to monotone miRNA expression in *Raphanobrassica* are complicated. Moreover, even miRNAs in a same miRNA family can display different regulation patterns. For example, miR156a was down-regulated in *Raphanobrassica,* but miR156b-3p was up-regulated, suggesting a difference in the regulation of expression levels between miRNAs and their target mRNAs. The miRNA expression mechanism is analogous to that in cold-stressed populus^34^ and the peach^46^. A previous study showed the indirect miRNAs modulatory effects of mRNAs^45^, and miRNA was also demonstrated to indirectly interact with mRNAs transport factor genes^47^. Furthermore, among the 178 related target genes, 31(17.41%) genes were non-additively expressed and significantly enriched in the terms ‘metabolic process’, ‘response to abiotic stimulus’ and ‘response to stress’ in *Raphanobrassica*. miRNA5658, which has the largest related non-additive genes in the present study, was reported to be significantly enriched in the terms ‘response to stress’ and ‘response to biotic and abiotic stress’ in *Ocimum basilicum*^42^ and significantly enriched in the term ‘metabolic process’ in *Raphanobrassica*. miRNA5658 might not only play particular roles in response to drought in plant regulating mechanism but also make a non-negligible elemental contribution to the regulation of biological metabolism. Thus, the mRNAs-miRNAs regulation pattern is a potential key regulator that is critical for the fitness and development of the tetraploid *Raphanobrassica.*

### Regulation of gene expression in inter-generic allopolyploidy may increase the potential of a defence response

A marketable study demonstrated that polyploidy enhances stress responses compared with diploidy^14^,^16^,^27^. Analysis of Arabidopsis gene expression suggests that the stress-related genes are conserved during polyploidization^48^. Chen^25^ observed that the genes *SCHLAFMUTZE* and *SCHNARCHZAPFEN* in the *AP2* TF family were inhibited by miR172 to regulate flowering time in Arabidopsis. Because overexpressing miR172 induced early flowering and organ mutations^49^, moderate inhibition could promote growth^50^. In the *Brassica* hexaploid^15^, the genes involved in the biological processes of ‘response to stimulus’ and ‘metabolic pathway’ were significantly up-regulated compared with its parents. In the present study, the related target genes involved in ‘response to stimulus’ and ‘metabolic pathway’ were differentially expressed in *Raphanobrassica* compared with its parents. For example, miR169, which is down-regulated under drought conditions, and its target gene *NFYA5* were up-regulated and contributed to strong drought resistance in Arabidopsis^51^, and these genes were up-regulated in *Raphanobrassica* compared with its parents. Bra015496, encoding a salt tolerance-related protein targeted by miR5256, was also up-regulated in *Raphanobrassica* and its parents.

The metabolic pathway plays an unneglectable role in the plant defence response. Under hyperthermia, ARR5 gene family shows a negative correlation with the plant defence mechanism^52^. In the present study, the transcription factor gene bra001641 of the homologous gene family ARR5-B was detected and implicated in the metabolic pathway. Remarkably, this gene is down-regulated in *Raphanobrassica* compared with its parent, reflecting a negative correlation; thus, an efficient defence mechanism may exist. The differentially expressed gene bra001641 was also non-additively expressed in the present study and showed maternal bias. These findings suggested that the regulation of non-additively expressed genes in *Raphanobrassica* might increase the defence response.

## Conclusion

The present study is the first to explore the data of mRNAs and miRNAs to identify genes and miRNA expression levels in the inter-generic hybrid tetraploid *Raphanobrassica* and its parents. Several mRNAs and miRNAs from *Raphanobrassica* and its parents that are involved in diverse pathways were detected. Non-additively expressed genes in the inter-generic hybrid tetraploid *Raphanobrassica* revealed some key pathways (‘antioxidant’, ‘auxiliary transport protein’ and ‘multi/-organism process’), which were different from non-additively expressed genes in the interspecific *Brassica* hexaploid^15^, providing informative results to detect the difference between inter-generic and interspecific polyploids. Interestingly, the ‘metabolic process’ pathway was significantly enriched both in non-additively expressed mRNAs/miRNAs (target genes) or related target genes from the miRNA-mRNA network in *Raphanobrassica* compared with its parents, and this pathway might play an important role in *Raphanobrassica.* The relationship between miRNAs and mRNAs is complex and dynamic, and further studies on the mRNA-mRNA network are required to deeply analyse the superiority of allopolyploids.

## Methods

### Plant materials

Plant materials included *Brassica oleracea* var. *alboglabra* (CC 2n = 18), *Raphanus sativus* (RR 2n = 18) and *Raphanobrassica* (RRCC 2n = 36). The amphidiploid *Raphanobrassica* (RRCC 2n = 36) was generated by crossing paternal cabbage mustard (*B. oleracea* var. *alboglabra*) with maternal radish (*R. sativus*) followed by chromosome doubling, and the plants were grown and self-pollinated in the field of Huazhong Agricultural University, China, in the sixth generation^39^. Immature leaves of *Raphanobrassica* and its parents were cut and immediately frozen in liquid nitrogen, then store them in ultra-low temperature freezers for later use.

### Library construction and high-throughput sequencing

The sequencing libraries were prepared using the Illumina RNA-seq kit as described by Xu *et al*.^28^. Total RNA from *Raphanobrassica* and its parents was extracted using TRIzol reagent (Invitrogen, Burlington, ON, Canada) and then RNase-free DNase I was used to avoid genomic DNA contamination. Dynabeads oligo (dT) (Dynal, Invitrogen) was performed to extract mRNA. The double-stranded cDNAs were synthesized using the reversed transcriptase (Superscript Double Stranded cDNA synthesized kit; Invitrogen) and random hexamer primers (Illumina, San Diego, CA, USA). Then the RNA-seq libraries were constructed by standard Illumna protocol. RNA quality was corrected on Agilent 2100 NanoDrop bioanalyzer. Only samples containing suitable standards (m ≥ 10 μg, c ≥ 200 ng/μL, OD_260/280_ ≥ 1.8) were accepted for further analysis^16^.

### mRNA sequencing data analysis and gene identification

To study the effect of ployploidization on gene expression in inter-generic synthetic tetraploid *Raphanobrassica*, we used the method of RNA-seq (modified) to compare the difference of gene expression between *Raphanobrassica* and its parents. After discarding the low quality raw reads, a total of 61,396,492 sequence reads (average length of 140 bp) were generated from cDNA libraries driven from *R. sativus, B. oleracea* var. *alboglabra*, and *Raphanobrassica.*

### Functional analysis and characterization of unigenes and DEGs

Gene function was annotated based on the following databases: Nr (NCBI non-redundant protein sequences); Nt (NCBI non-redundant nucleotide sequences); GO (Gene Ontology), KO (KEGG Ontholog database). Plant Transcription Factor Database (http://brassicadb.org/brad/index.php) was used for identification and classification of transcriptional factors. For further study of the significance variation of the detected genes in biological processes/pathway, GO functional classes (http://www.geneontology.org/) and BLAST2GO (version 2.3.5) (http://www.blast2go.org/) were used to precisely define relevance genes. GO enrichment analysis was identified with the BinGO plugin Cytoscape. Kyoto Encyclopedia of Genes and Genomes (KEGG) is a database linking genomic information with higher order functional information by mapping them to reference canonical pathways (http://www.genome.ad.jp/kegg/). KEGG pathway enrichment analysis was done using KOBAS software (KOBAS, Surrey, UK).

### Identification of non-additive expressed genes

To determine non-additively expressed genes, we compared gene expression levels in *Raphanobrassica* with the average genes expression values in the parents (MPVs). If the value of a gene in *Raphanobrassica* showed at least a two-fold change and p < 0.05 relative to the MPV, then the gene was regarded as non-additive expression^37^.

### Analysis of differential expressed gene

Differential expressed genes were performed using the R package DEGseq^53^. Genes with log_2_Ratio ≥ 1, 0.05 were defined differentially expressed genes (DEG).

### Quantitative RT-PCR validation for mRNAs

Total RNA was fetched from young leaves of *R. sativus, B. oleracea* var. *alboglabra* and tetraploid *Raphanobrassica*. Quantitative PCR was used to quantify the significantly differentially expressed mRNAs, ten mRNAs were obtained from high through-put sequencing, and qPCR assays were carried out using SYBR kit under the LightCycler Real-Time PCR System with the parameters: 30 s at 95 °C, 40 cycles at 95 °C for 5 s, 30 s at 60 °C. Triplicates of each reaction were performed as the mean ± SD. CT method were used to calculate comparative the relative fold change values. The detail primers used in all quantitative qRT-PCR are given in Table 3.

### Small RNA library preparation

For sRNA library construction, young leaves of *R. sativus, B. oleracea var. olablagra* and *Raphanobrassica* were separately extracted using Trizol reagent (Invitrogen, USA). Then use RNase-free DNase I (Fermentas, Canada) to remove the residual DNA. Sequencing libraries were generated using Illumina high-throughput sequencing technology at Beijing Genomics Institute (BGI).

### Analysis of small RNA sequencing data

Illumina high-throughput sequencing technology was employed to study the variation of small RNAs and their potential roles in gene regulation *Raphanobrassica* and its parents. After cluster generation, the library preparations were sequenced on Illumina Hiseq 2000 platform and based on BGI small RNA pipeline, and the adaptor sequences and lower quality reads were removed. Then the 18–30 nt RNAs were aligned to the *B. rapa* genome using SOAP software^34^ to identify the repeat-associated sRNAs and to assess the expression of sRNAs. The cellular structural RNAs which include tRNA, scRNA, snoRNA or snRNA, were removed comparison with the NCBI nucleotide database and Rfam RNA family database. The miRNAs were indentified using miRBase. In order to realize the differential expression of miRNAs, the abundance of miRNAs in three libraries was normalized as values to compare between each two libraries and were calculated in the form of fold changes (fold change = log_2_). In addition, p-value was used to be a previously described formula^54^. The gene function includes biological process, cellular component and molecular function which were analyzed for the predicted target genes.

### Differential expression analysis of miRNAs

Differentially expressd miRNAs between *Raphanobrassica* and its parents were performed using the DEGseq software^55^. P-value was adjusted-using q-value. Q-value < 0.01 and log_2_-fold change >1 was set as the threshold significantly differential expression by default.

### Identification of ELD-miRNAs between *Raphphanobrassica* and its parents

In order to understand how hybridization and polyploidy regulate miRNAs expression, we compared miRNAs expression in *Raphanobrassica* with the mid-parent value (MPV) from parents species to deem the ELD-miRNAs (If the value of a miRNAs showed at least a twofold change and 0.05 compared with MPV), the others were considered to be additive miRNAs.

### MiRNA target gene prediction

Prediction of conserved miRNAs target genes was performed by psRobot_tarscripts in psRobot^56^.

### Quantitative RT-PCR validation for miRNAs

Total RNA was fetched from young leaves of *R. sativus, B. oleracea* var. *alboglabra* and tetraploid *Raphanobrassica*. Stem-loop RT-PCR was used to validate miRNA expression, and the miRNA-specific stem-loop primers were assigned as Chen^39^. qRT-PCR was performed in triplicate combine with the SYBR kit, and the samples were amplified using the LightCycler Real-Time PCR System under the process: 30 s at 95 °C, 40 cycles at 95 °C for 10 s, 30 s at 56 °C, 15 s at 72 °C. Fold changes in miRNAs of different samples were calculated by CT method. The primers were detail listed in Table 3.

### Correlation analysis of miRNA and mRNA

In order to build the miRNA-mRNA interaction network, including positive and negative relationships between mRNA and miRNA, we used an in-house method to construct miRNA-mRNA network. We integrated the expression data with mRNAs, and the main concern was negatively correlated miRNAs and mRNAs. We defined a predicted miRNAs target gene as related target gene if its expression pattern was contrary with the miRNAs, for example, of all miRNAs up-regulated in a *Raphanobrassica*, the down-regulated expression target gene we call relevance target genes, the others we called non-related target genes; contrapose down-regulated miRNAs in *Raphanobrassica*, the target genes which up-regulated we call related target genes, the others called non-related target genes.

## Additional Information

**How to cite this article**: Ye, B. *et al*. Correlation analysis of the mRNA and miRNA expression profiles in the nascent synthetic allotetraploid *Raphanobrassica. Sci. Rep.* **6**, 37416; doi: 10.1038/srep37416 (2016).

**Publisher’s note**: Springer Nature remains neutral with regard to jurisdictional claims in published maps and institutional affiliations.

## Supplementary Material

Supplementary Information

## Figures and Tables

**Table 1:** Number of reads matched to *B. rapa* genome from *R. sativus, B. alboglabra* and *Raphanobrassia*, respectively.

Species	Total reads	Total mapped reads	Unique match	Muti-position match	Unmapped reads
*R. sativus*	20,041,964 (100.00%)	18,311,829 (91.37%)	16,840,427 (84.03%)	1,471,402 (7.34%)	1,730,135 (8.63%)
*B. alboglabra*	21,557,455 (100.00%)	19,821,630 (91.95%)	18,723,004 (86.85%)	1,098,626 (5.10%)	1,735,825 (8.05%)
*Raphanobrassica*	19,797,073 (100.00%)	18,198,114 (91.92%)	17,068,251 (86.22%)	1,129,863 (5.71%)	1,598,959 (8.08%)

**Table 2:** Reads quantity distribution in all detected miRNAs.

Reads quantity	*R. sativus*	*B. alboglabra*	*Raphanobrassica*	Percentage (%)
<100	121	129	119	43.53
100–500	52	65	57	20.64
500–1,000	25	58	21	12.33
1000–5,000	45	42	11	16.13
5000–10,000	8	9	49	7.82
>10,000	27	16	21	7.59
Total	277	274	278	100

**Table 3:** Primer sequence (5′-3′) design of mRNAs and miRNAs for qRT-PCR validation.

Primer name	Forward	Reverse
Bra005425	GGACACCGCTGGTCTATC	GCGACTCTGTAACCCTCAA
Bra028174	CGGATACTACGATGGACG	AGGCGATGAAACTGATGC
Bra008661	GGCAGACAACAAGCAAAG	CAGCAGAGGTAGCAGCATC
Bra030182	GTTCAAGGCTGGCTCACA	GCAAGGTTCTCCAAGGGT
Bra025297	ACCAGACCGTCCCAAGTA	TTCCCGAGGTAATCAAGC
Bra020868	GTGCGGTTCTGTCTCCTG	AGTGACGCTCATCGGTAG
Bra025431	GAGTTGGAGCACGGATTT	GGACCTGACGGTTGTTGT
Bra013183	CCGTCAAGTCCACTCCTCAAA	CCGTCAAGTCCACTCCTCAAA
Bra013576	GATGATGGTGGCGATGCT	TTGTGGTCGGGAAGGTAA
Bra016047	CACCACCAACATCATCAGC	GGCGGTAGGAAAGTAGTAGC
miRNA216	GCCGCTGCAGCACCATTAAGA	GTGCAGGGTCCGAGGT
miRNA151	GCCGCTCTTGCATATCTTAGG	GTGCAGGGTCCGAGGT
miRNA107	GCCGCTGGAATGTTGTCTGGC	GTGCAGGGTCCGAGGT
miRNA109	GCCGCTGCGTATGAGGAGCCA	GTGCAGGGTCCGAGGT
miRNA40	GCCGCTGCAGCACCATCAAGA	GTGCAGGGTCCGAGGT
miRNA54	GCCGCTGCGTATGAGGAGCCA	GTGCAGGGTCCGAGGT
miRNA69	GCCGCTTGATCTTGTGTTGTG	GTGCAGGGTCCGAGGT
miRNA185	GCCGCTGGACTGTTGTCTGGC	GTGCAGGGTCCGAGGT
miRNA100	GCCGCTTTTGTTCATGACTGC	GTGCAGGGTCCGAGGT
