# Supplementary material for: Correlation analysis of the mRNA and miRNA expression profiles in the nascent synthetic allotetraploid Raphanobrassica
Source: Sci Rep. 2016 Nov 22;6:37416. doi: 10.1038/srep37416 (PMC5118723; doi:10.1038/srep37416)
Supplement: Supplementary Information [file srep37416-s1.doc]

Correlation analysis of the mRNA and miRNA expression profiles in the nascent synthetic allotetraploid *Raphanobrassica*

Bingyuan Ye, Ruihua Wang & Jianbo Wang*

State Key Laboratory of Hybrid Rice, College of Life Sciences, Wuhan University, Wuhan 430072, China

Corresponding author: Dr. Jianbo Wang

Tel: +86-27-68752213

Fax: +86-27-68752213

E-mail: jbwang@whu.edu.cn

**Fig. S1** The different read coverage of genes in three libraries. Different colors indicated the genes with different read coverage. The capital letters A, B, and C in brackets refer to libraries of *R. sativus*, *B*. *oleracea* var. *alboglabra*, and *Raphanobrassica,* respectively.


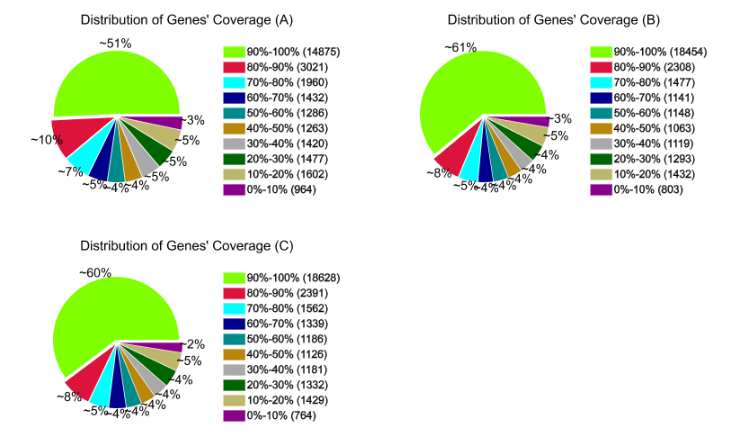


**Fig. S2** Gene ontology (GO) annotations of all detected genes. The histogram shows the result of classifying 28,314 genes to the secondary classification of GO terms. The x-axis indicates the number of genes in a functional term.


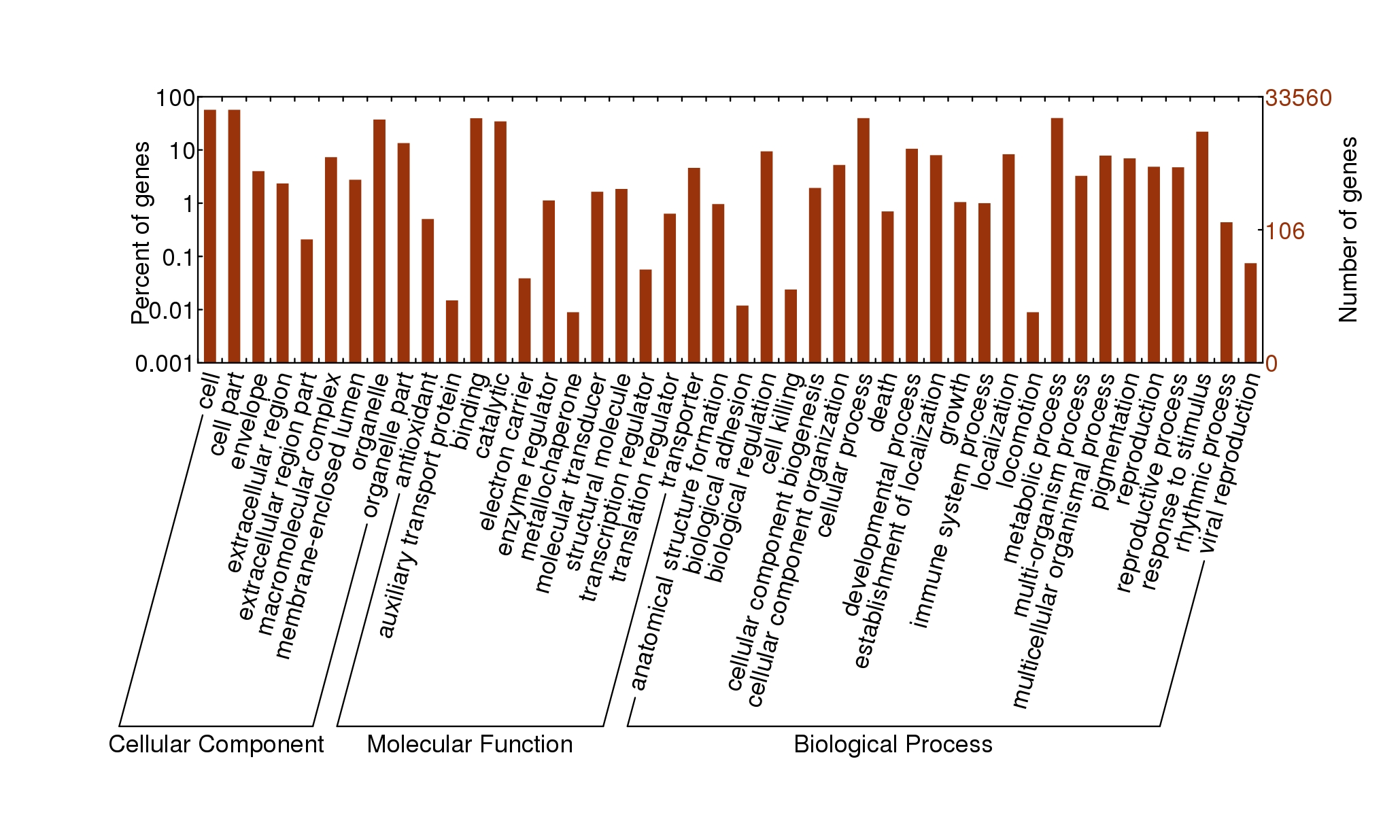


**Fig. S3** There were 3,387 genes in 63 transcription factor families and the highest 15 families have been named.


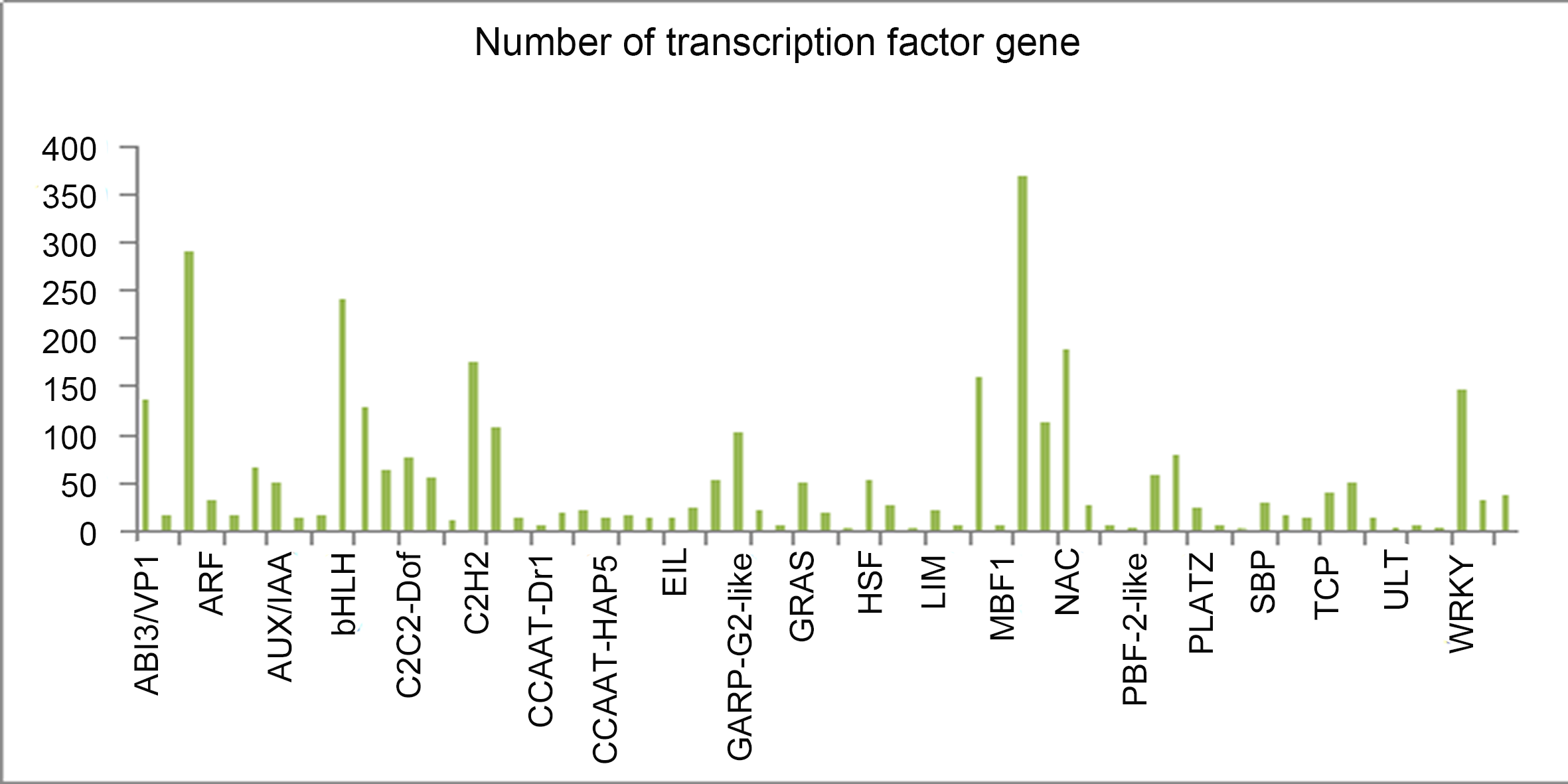


**Fig. S4a** GO annotation of ELD mRNAs. (a) ELD-a mRNAs annotation; (b) ELD-b mRNAs annotation.


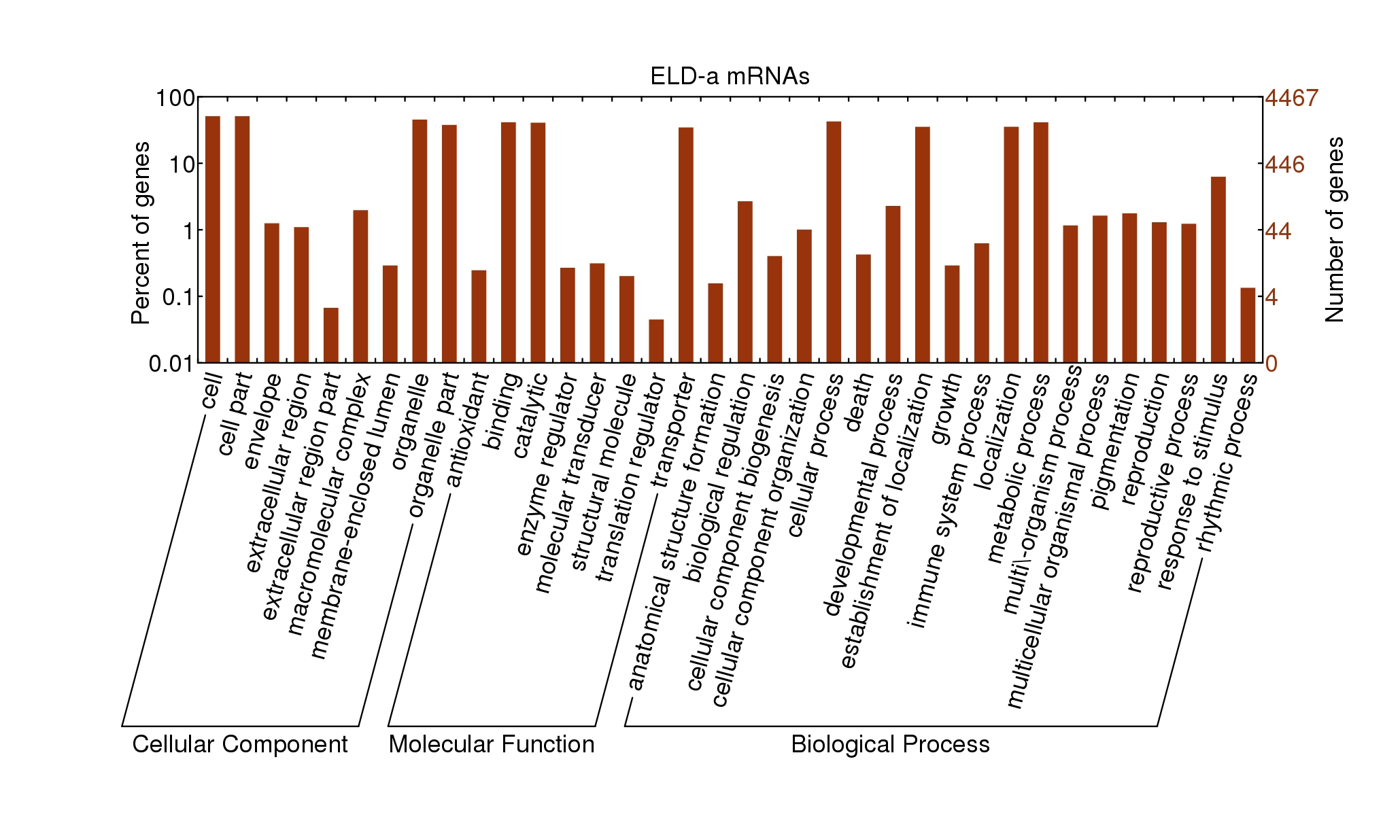


**Fig. S4b** GO annotation of ELD mRNAs. (a) ELD-a mRNAs annotation; (b) ELD-b mRNAs annotation.


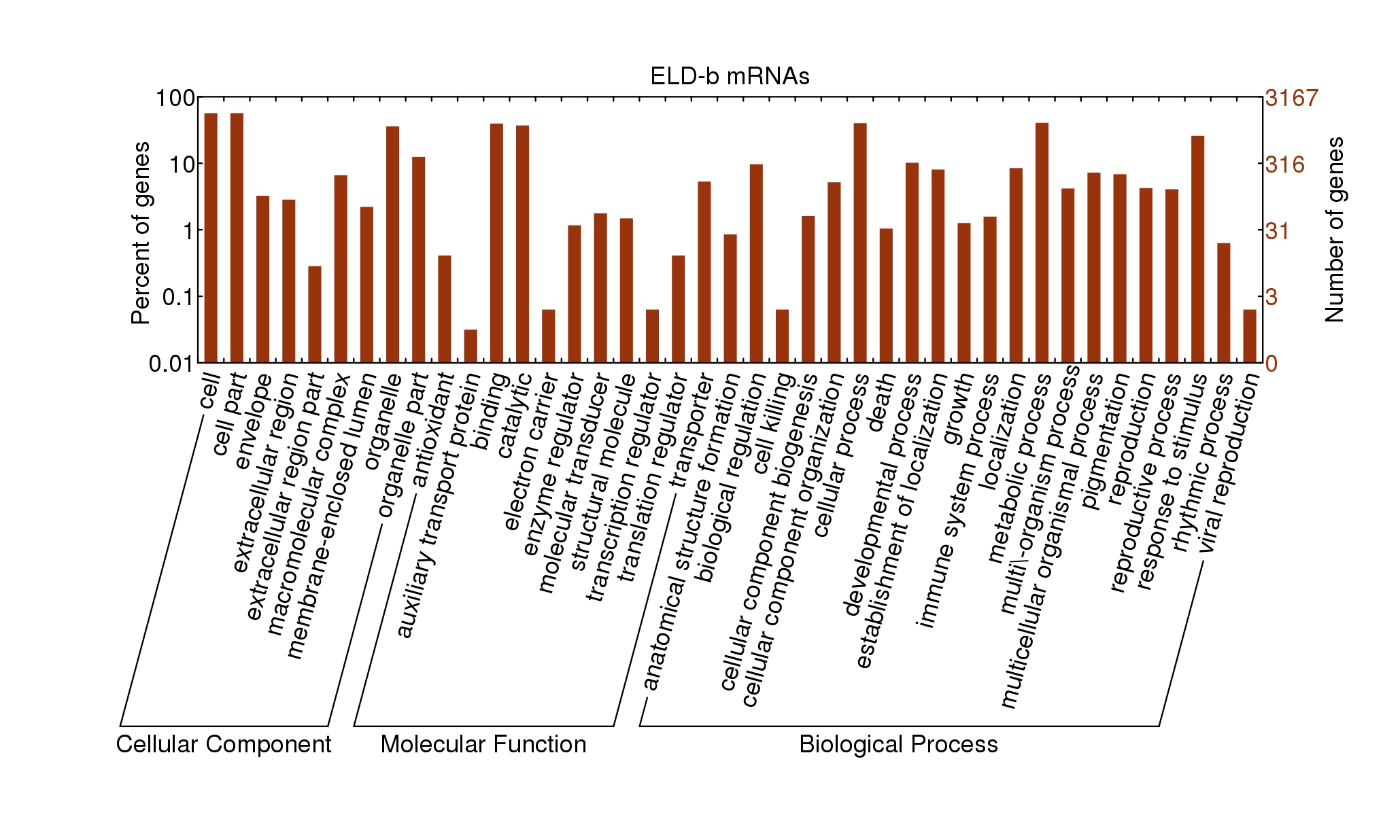


**Fig. S5** Venn diagram showed miRNAs distribution in *Raphanobrassica* and its parents. A, *Raphanus sativus;* B*, Brassica oleracea* var. *alboglabra;* C, *Raphanobrassica*.


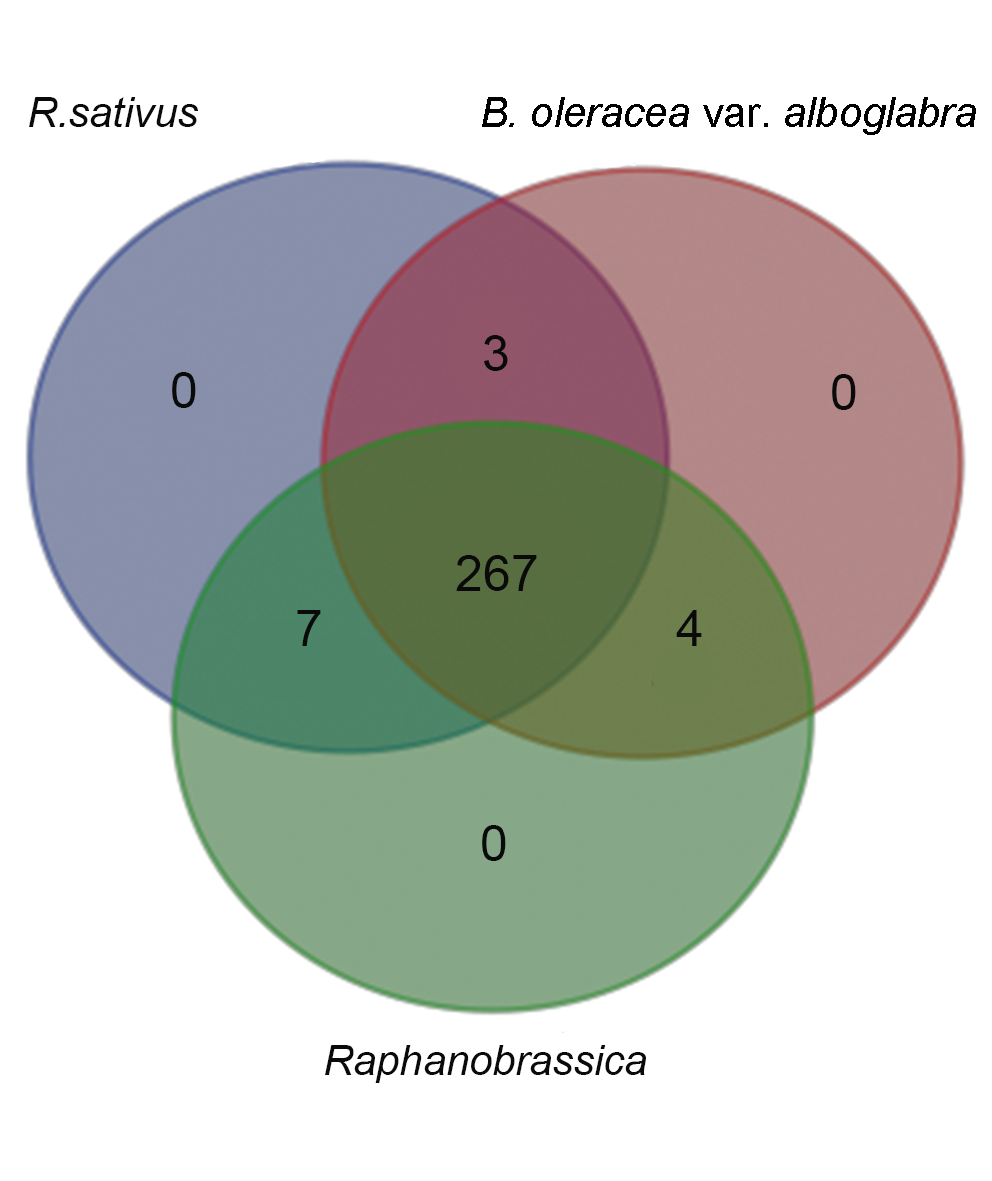


**Fig. S6** GO annotation of miRNAs target genes between *Raphanobrassica*/*R. sativus* and *Raphanobrassica*/*B. oleracea* var. *alboglabra*. A, *Raphanus sativus;* B*, Brassica oleracea* var. *alboglabra;* C, *Raphanobrassica*.


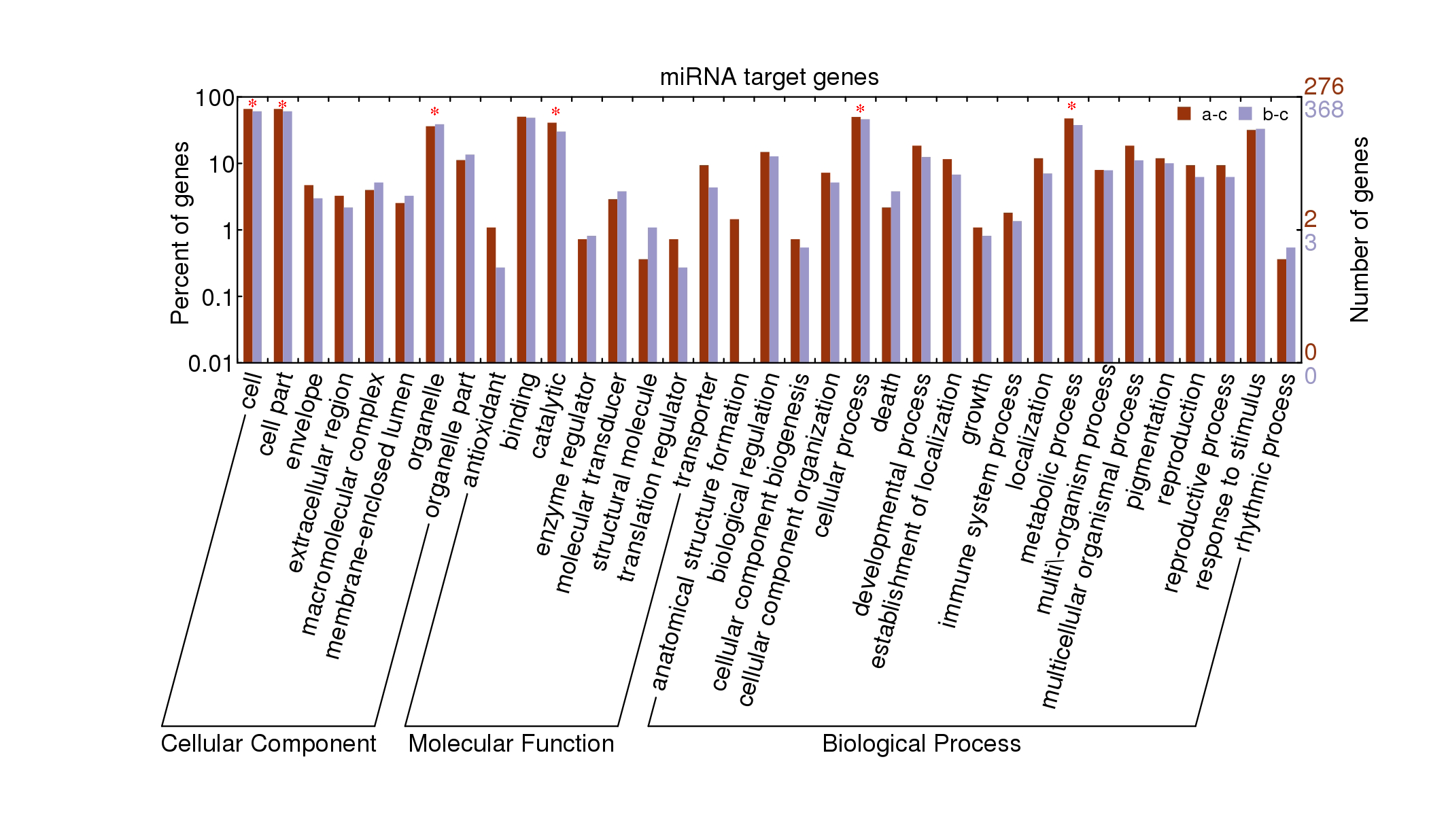


**Fig. S7** GO annotation of non-additive miRNA target genes.


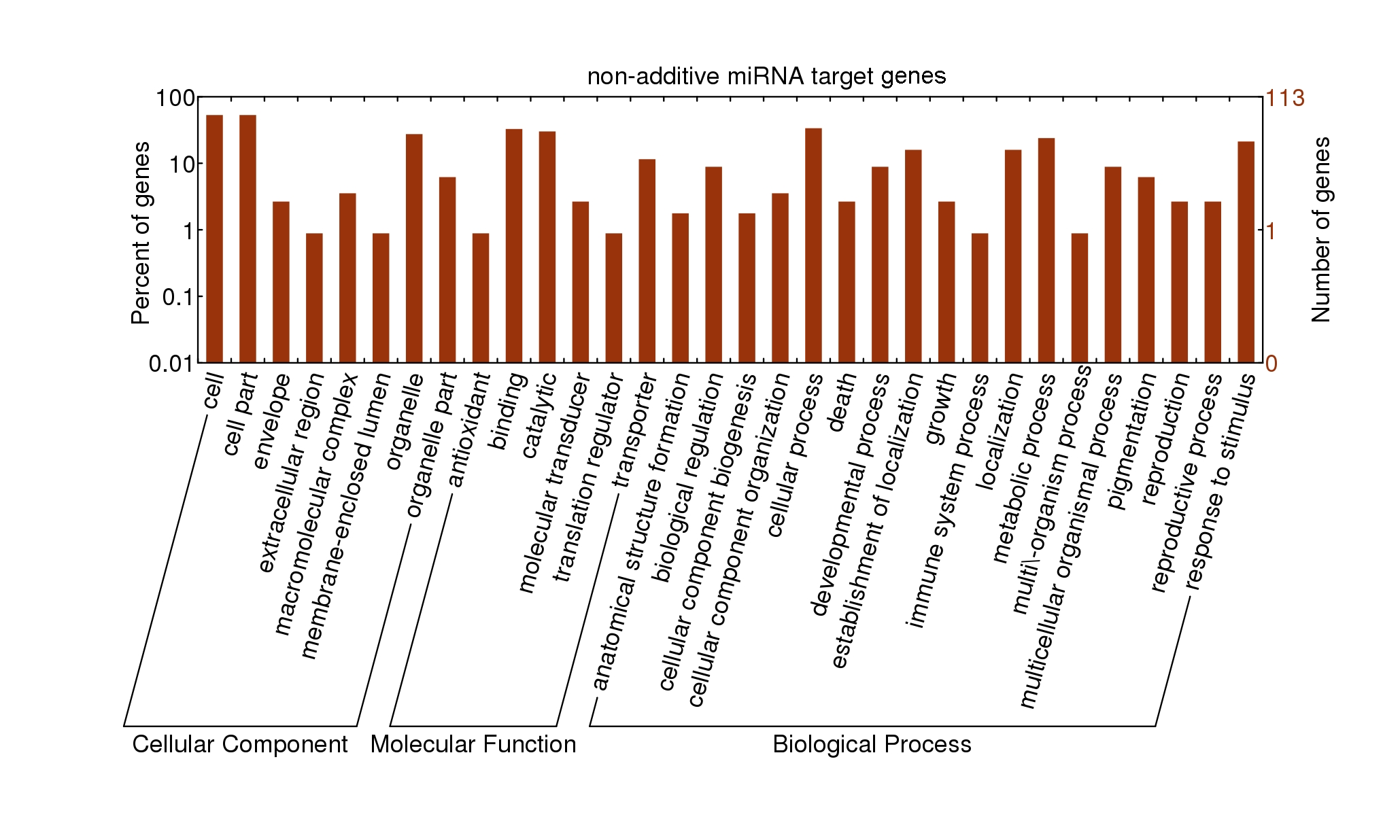


**Fig. S8** GO annotation non-additive related target gene.


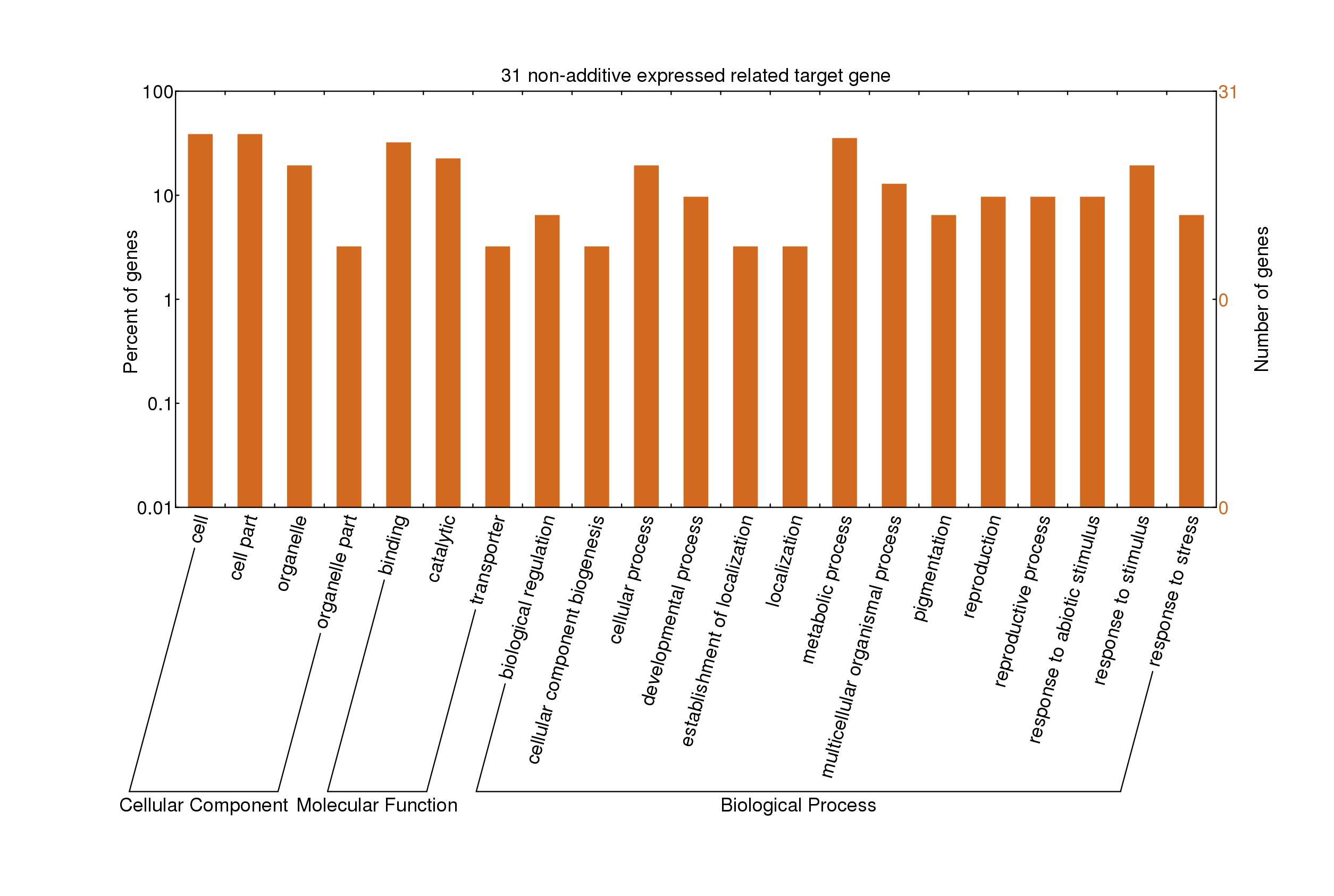


**Table S1.Gene distribution of the ten highest KEGG pathways.** According to the enriched gene numbers, ten significantly enriched pathways were detected from 127 KEGG pathways, the average value of gene expression is base on the mean value of *Raphanobrassica* and its parents *R. sativus*, *B. oleracea*var. *Olbaglabra*.

| **Pathway name** | **Gene numbers** | **Average expression** |
| --- | --- | --- |
| Metabolic pathways | 1,716 | 115,641.64 |
| Biosynthesis of secondary metabolites | 651 | 3,611.01 |
| Plant-pathogen interaction | 589 | 5,109.00 |
| Phenylpropanoid biosynthesis | 195 | 4,975.68 |
| Ribosome | 180 | 24,613.11 |
| Starch and sucrose metabolism | 176 | 5,045.07 |
| Protein processing in endoplasmic reticulum | 161 | 4,293.91 |
| RNA transport | 157 | 2,972.54 |
| Stilbenoid, diarylheptanoid and gingerol biosynthesis | 147 | 2,780.78 |

**Table S2.Known miRNAs and target genes prediction. The table showed the miRNA and their target gene both single and co-existed in *R. sativus*, *B. oleracea*var. *Olbaglabra* and *Raphanobrassica*.**

| | **Species** | **miRNA number** | **Target gene number** | **Target location number** | | --- | --- | --- | --- | | *R. sativus* | 182 | 1,103 | 1,321 | | *R. sativus-B. alboglabra* | 114 | 699 | 831 | | *R. sativus-Raphanobrassica* | 85 | 394 | 498 | | *B. alboglabra* | 179 | 1,098 | 1,316 | | *B.alboglabra-Raphanobrassica* | 74 | 478 | 513 | | *Raphanobrassica* | 182 | 1,102 | 1,319 | |
| --- | --- | --- | --- | --- | --- | --- | --- | --- | --- | --- | --- | --- | --- | --- | --- | --- | --- | --- | --- | --- | --- | --- | --- | --- | --- | --- | --- | --- |
